# Mother’s perception of size at birth is a weak predictor of low birth weight: Evidence from Nepal Demographic and Health Survey

**DOI:** 10.1371/journal.pone.0280788

**Published:** 2023-01-24

**Authors:** Pawan Acharya, Suyasha Adhikari, Tara Ballav Adhikari

**Affiliations:** 1 Nepal Development Society, Bharatpur, Chitwan, Nepal; 2 Doctoral Candidate in Epidemiology, Department of Biostatistics and Epidemiology, Hudson College of Public Health, The University of Oklahoma Health Sciences Center (OUHSC), Oklahoma City, OK, United States of America; 3 Nepal Health Frontiers, Kathmandu, Nepal; 4 Section for Environment, Occupation & Health, Department of Public Health, Aarhus University, Aarhus, Denmark; Johns Hopkins University Bloomberg School of Public Health, UNITED STATES

## Abstract

Birth weight is a consistent predictor of morbidity and survivability in infancy and later life. This study aims to assess the accuracy of the mother’s perception of size at birth to predict low birth weight(LBW). This study used data from Nepal Demographic and Health Survey (NDHS 2016). Information about 5060 mother pairs was obtained from the NDHS dataset. However, birth weight data were available for 3095 children, and therefore they were only included in the further analysis. The predictive accuracy of the mother’s perception of size at birth to predict LBW was measured by sensitivity, specificity, positive predictive value, and negative predictive value. Factors associated with the discordance among the mother’s perception of birth size and birth weight were calculated using multinomial logistic regression analysis. The mother’s perception of birth size had low sensitivity (62%) and positive predictive value (46.7%) but high specificity (90.1%) and negative predictive value (94.4%) to predict the LBW. The overall agreement between birth weight(<2500gram vs > = 2500 grams) and the mother’s perceived size at birth (small vs average or above average) was 86% (Kappa = 0.45, 95%CI: 0.40–0.51), which is composed of a higher share of the agreement to identify non-LBW babies(79%) and a low share to identify LBW babies (7%). Among the five categories of mothers’ perception of size at birth and birth weight, the agreement was 67.2% (Kappa = 0.29, 95% CI: 0.26–0.33). Education status, ethnicity, multiple births, and sex of the newborn child were significantly associated with the discordance between the mother’s perceived size at birth and birth weight. A moderate agreement was found among the mother’s perception of birth size and birth weight. Mothers were more likely to correctly identify non-LBW babies compared to LBW babies based on their perception of size at birth. Efforts should be intensified to promote the practice of weighing the baby at birth.

## Introduction

Birth weight is a consistent predictor of morbidity and survivability in infancy, early childhood, and later life [1, 2]. The World Health Organization (WHO) defines birth weight <2500 grams as low birth weight (LBW) regardless of gestational age [3]. Globally, the prevalence of LBW is estimated to be 14.6%, with uncertainties ranging from 12.4–17.1%. About 91% of the total LBW babies are born in low and middle-income countries. Southern Asia and sub-Saharan Africa share about 48% and 24% global burden of LBW respectively [4].

Identification of LBW is key to ensuring the special care needed by these infants. A major challenge in dealing with the LBW burden among developing countries is not having birth weight data. Globally, about 48% of infants are not weighed at birth, with estimates ranging from as low as 10% in Latin America and the Caribbean to as high as 66% in South Asia [3, 5].

The mother’s perception of the baby’s size at birth is associated with the sex of the newborn, the child’s vital status at the time of the survey, and contextual factors like the neighborhood [6]. Mothers’ perception of the size of their baby at birth has also been associated with breastfeeding initiation [7–9] and child nutrition status [10]. These facts highlight the differential childcare practices according to the mother’s perception of size at birth.

Nationally representative surveys, like the Demographic and Health Surveys (DHS) and Multiple Cluster Indicator Surveys (MCIS), regularly collect information about the mother’s perceived size at birth, but a significant proportion of babies in these surveys have missing birth weight data. Since the information regarding birth weight is not available for a considerable proportion of children, the mother’s perception of the baby’s size at birth is used as the proxy of birth weight.

Studies have suggested using the perceived size at birth as a proxy for birth weight with caution as it doesn’t correctly predict birth weight [6, 11, 12]. Also, the concordance between the mother’s perception of size at birth and actual birth weight is influenced by various contextual, socio-economic, and bio-demographic factors of the family, mother, and newborn [6, 11, 12].

This study aims to harness the publicly available data from the Nepal Demographic and Health Survey (NDHS) to assess the accuracy of a mother’s perception of the size at birth to predict the birth weight among newborn babies, with a particular focus on the predictive accuracy to detect LBW.

## Materials and methods

### Data source and context

This study represents the data from the NDHS 2016. NDHS is a nationally representative sample survey conducted in every five years interval. NDHS employs a two-stage cluster sample survey methodology. Details about the methodology and survey deployment can be found in the survey report [13].

The sampling frame of NDHS is the women of reproductive age 15–49 years in the selected households. The survey’s child datasets were used for this analysis. In the survey database information was available for 5060 (unweighted number: 5038) children born five years before the surveys. However, only about 61% of children had recorded birth weight in grams. A total of 3095 (unweighted number: 3122) children who had both measurements for the mother’s perception at birth and birth weight were included in the final analysis.

### Outcome variable

#### Mother’s perceived size at birth

During the survey, mothers were asked *“When (NAME) was born*, *was (NAME) very large*, *larger than average*, *average*, *smaller than average*, *or very small*?*”* The response was recorded as very large, larger than average, average, smaller than average, very small, and don’t know. To analyze the predictive accuracy of this variable to detect the low birth weight, a separate variable was also created and labeled as “Perceived low size at birth”, with two categories- Small (combined smaller than average and very small) and Average or larger (combined average, larger than average and very large).

#### Birth weight

During the survey, mothers were asked if the baby was weighed at birth. If the baby was weighed, they were further asked *“How much did (NAME) weigh*?*”* The response was recorded based on the mother’s recall or the actual record from the record card. The information was recorded in the kilogram and up to three characters after the decimal. Later, the values were converted to grams. An indicator variable was created for LBW following the WHO criteria, LBW = 1 if the birth weight was <2500grams [3].

The birth weight was normalized and categorized into five categories based on the standard deviation (>2 SD = very large; 1 to 2 SD = larger than average; -1 to 1 SD = average, -1 to -2 SD = smaller than average, and < -2 SD very small). This normalized variable was used to estimate the agreement between the mother’s perception of the birth size and measured birth weight.

Agreement between the mother’s perceived size and measured birth weight was measured into three categories- concordant (mother’s perceived size = normalized birth weight category), underestimated (mothers’ perception < normalized body weight), and overestimated (mothers’ perception > normalized body weight).

### Explanatory variables

Covariates were selected based on the literature review and their likely clinical significance in the multivariable analysis. Place of residence, Ethnicity, Wealth quantile, Mother’s age at delivery, Mother’s education, Marital status, Antenatal care (ANC) visits, Place of delivery, and Delivery by cesarean section (CS) were included in the analysis. Also, child-specific factors like Multiple births, Sex of the child, Birth order, Preceding birth interval, and Pregnancy intentness were included in the analysis. Ethnicity was further categorized into three categories: Advantaged (including Brahmin/Chhetri cast), relatively disadvantaged (including Janajatis, Muslims, and Other ethnic minorities), and disadvantaged (including Dalits). The wealth quintile was derived from the wealth index. The wealth index was calculated by principal component analysis using household assets and was categorized into five categories comprising 20 percent of the population in each category [14].

### Statistical analysis

The sample distribution of the total under-five children included in the NDHS survey and also the proportion of babies weighed at birth were tabulated according to the background characteristics. The likelihood of being weighed at birth in terms of odds ratio (OR) with a respective confidence interval (CI) was calculated using logistic regression analysis. Furthermore, the sampling distribution of children who were weighed at birth was calculated separately. One-way ANOVA was used to test if there was a significant difference in the mean birth weights of different perceived categories. The predictive accuracy of the mother’s perception of birth size to detect the LBW babies was estimated by calculating sensitivity, specificity, positive predictive value (PPV), and negative predictive value (NPV) with respective 95% CI.

Similarly, the proportion of the different categories of agreement between mothers’ perception of size at birth and actual birth weight was calculated according to the background characteristics. The association between the characteristics and agreement was assessed in the descriptive analysis using the chi-square test (Table 1).

**Table 1:** Background characteristics of mother and child with non-missing birth weight, NDHS 2016 (n = 3095).

			Agreement between weight and perception						
Characteristics	Sample		Concordant		Underestimate		Overestimate		p-value
	n	%	Row %	95% CI	Row %	95% CI	Row %	95% CI	
**Place of residence**									p = 0.133
Urban	1953	63.11	67.99	[65.36,70.52]	16.98	[14.87,19.34]	15.02	[13.29,16.93]	
Rural	1142	36.89	65.28	[62.22,68.21]	16.55	[14.39,18.97]	18.17	[15.84,20.76]	
**Mothers education**									p = 0.398
No educcation	689	22.27	65.58	[61.37,69.56]	19	[15.74,22.75]	15.42	[12.67,18.64]	
Primary	558	18.03	68.02	[63.52,72.20]	14.32	[11.43,17.79]	17.67	[14.34,21.57]	
Secondary or above	1848	59.7	67.21	[64.55,69.76]	16.77	[14.67,19.11]	16.02	[14.21,18.01]	
**Marital status**									p = 0.318
Single or unmarried	28	0.92	63.75	[43.23,80.25]	27.12	[13.01,48.08]	9.13	[2.46,28.55]	
Married	3067	99.08	67.02	[65.02,68.97]	16.73	[15.14,18.44]	16.25	[14.83,17.78]	
**Ethnicity**									p<0.001
Advantaged	1041	33.64	64	[60.50,67.36]	18.68	[15.83,21.91]	17.32	[14.98,19.93]	
Relatively disadvantaged	1698	54.85	70.34	[67.70,72.85]	13.93	[12.10,15.99]	15.73	[13.80,17.87]	
Disadvantaged	356	11.51	59.78	[53.67,65.60]	25.18	[19.82,31.43]	15.04	[11.48,19.45]	
**Wealth quintile**									p = 0.012
Poorest	450	14.54	64.45	[60.05,68.62]	19.08	[15.75,22.93]	16.47	[13.44,20.02]	
Poorer	542	17.52	68.12	[63.63,72.30]	16.66	[13.57,20.28]	15.23	[12.07,19.03]	
Middle	682	22.02	65.63	[61.60,69.45]	15.32	[12.62,18.48]	19.05	[15.99,22.53]	
Richer	742	23.98	62.83	[58.37,67.07]	20.09	[16.46,24.29]	17.08	[14.17,20.45]	
Richest	679	21.94	73.7	[68.82,78.05]	13.41	[9.87,17.95]	12.9	[10.15,16.26]	
**Mother’s age at delivery**									p = 0.973
<20 years	848	27.41	66.63	[62.99,70.09]	17.06	[14.46,20.01]	16.31	[13.69,19.32]	
20 or more	2247	72.59	67.13	[64.73,69.44]	16.74	[14.82,18.84]	16.14	[14.49,17.93]	
**Four ANC visit**									p = 0.108
No	977	31.58	64.8	[61.20,68.24]	19.27	[16.43,22.47]	15.93	[13.56,18.62]	
4 or more	2118	68.42	68	[65.58,70.33]	15.7	[13.84,17.75]	16.3	[14.58,18.18]	
**Place of delivery**									p = 0.982
Non institutional	496	16.03	67.15	[62.58,71.42]	16.97	[13.83,20.65]	15.88	[12.69,19.68]	
Institutional	2599	83.97	66.96	[64.74,69.11]	16.8	[15.03,18.72]	16.24	[14.69,17.92]	
**Delivery by caesarian section**									p = 0.884
No CS	2654	85.76	67.18	[65.06,69.23]	16.8	[15.14,18.60]	16.02	[14.53,17.64]	
CS delivery	441	14.24	65.88	[59.93,71.36]	16.98	[12.62,22.47]	17.14	[13.30,21.80]	
**Child is twin**									p<0.001
single birth	3057	98.76	67.36	[65.35,69.31]	16.41	[14.83,18.12]	16.23	[14.81,17.76]	
1st of multiple	19	0.62	28.82	[13.63,50.96]	57.44	[36.08,76.33]	13.74	[5.05,32.31]	
2nd of multiple	19	0.62	46.75	[26.68,67.93]	42.19	[22.79,64.35]	11.06	[3.33,30.94]	
**Sex of child**									p = 0.089
Male	1649	53.29	65.64	[62.97,68.22]	16.54	[14.55,18.76]	17.81	[15.85,19.97]	
Female	1446	46.71	68.53	[65.50,71.42]	17.15	[14.72,19.87]	14.32	[12.38,16.52]	
**Birth order of child**									p = 0.930
First	1548	50.02	67.38	[64.47,70.17]	16.15	[13.93,18.65]	16.46	[14.42,18.74]	
Second	911	29.42	66.81	[63.04,70.38]	17.49	[14.53,20.90]	15.7	[13.26,18.48]	
Third or more	636	20.56	66.29	[62.23,70.13]	17.51	[14.55,20.93]	16.19	[13.37,19.48]	
**Preceding birth space<24 months**									p = 0.393
No	2812	90.87	66.85	[64.74,68.89]	16.66	[15.00,18.47]	16.49	[15.00,18.10]	
<24 months	283	9.13	68.42	[62.07,74.15]	18.45	[13.89,24.11]	13.13	[9.32,18.18]	
**Unintended pregnancy**									p = 0.307
Wanted	2559	82.67	67.44	[65.25,69.57]	16.27	[14.55,18.15]	16.29	[14.73,17.97]	
Unwanted	536	17.33	64.84	[59.95,69.45]	19.47	[15.71,23.87]	15.69	[12.55,19.43]	
**Total**	**3095**	**100**	**67.07**	**[65.08,69.01]**	**16.85**	**[15.26,18.56]**	**16.08**	**[14.67,17.60]**	

CI: Confidence Interval.

NDHS: Nepal Demographic and Health Survey.

The reliability of the mother’s perception to predict birth weight was measured by kappa statistics separately–to predict the LBW (two categories), and also to detect birth weight (5 categories of normalized birth weight). Kappa statistics with a 95% confidence interval were calculated together with the percentage of the expected and actual agreement [15, 16].

A multivariable multinomial logistic regression analysis [17] was used to assess the association between background characteristics and agreement between the mother’s perceived size at birth and LBW. The outcome variable consisted of three categories and the category “concordant” was considered the base category. Odds ratios (OR) and 95% confidence interval were calculated including clinically and socially relevant variables listed in Table 1 [11, 18].

Data analysis was performed using the statistical software STATA 16 using the survey analysis technique. Cluster survey design and individual sample weight were accompanied by the “***svy”*** command in Stata. A p-value <0.05 was considered statistically significant throughout the analysis.

## Results

### Characteristics of the study population

Among the total 5060 under-five year children in the dataset, only 3095 (61.2%) children had information about birth weight.

Among 3095 mother-child pairs who provided information on the birth weight (unweighted sample size = 3122), about 36.9% were from the rural area, about 22.3% of mothers had no formal education, and about 99% were married. Similarly, about 14.5% belonged to the poorest wealth quantile, while about 22% belonged to the richest wealth quantile. Similarly, about 27.4% of mothers were below 20 years of age at their recent delivery. About 14.2% had a cesarean delivery and about 1.2% of mothers gave birth to twin children (Table 1).

Only about 17.8% of the actual birth weight data were obtained from the written card, and the overwhelming majority (82.2%) of birth weight data was based on the mother’s recall. About 3.7% of mothers reported that their baby was very large size at birth and about 4.4% were very small at birth. A majority, 65.9%, reported that their baby was average size at birth (S1 Table).

Detailed information on the distribution of overall 5060 mother-child pairs with the odds of having weighed at birth is included in the S2 Table.

### Potential measurement error in birth weight data

**Fig 1:**
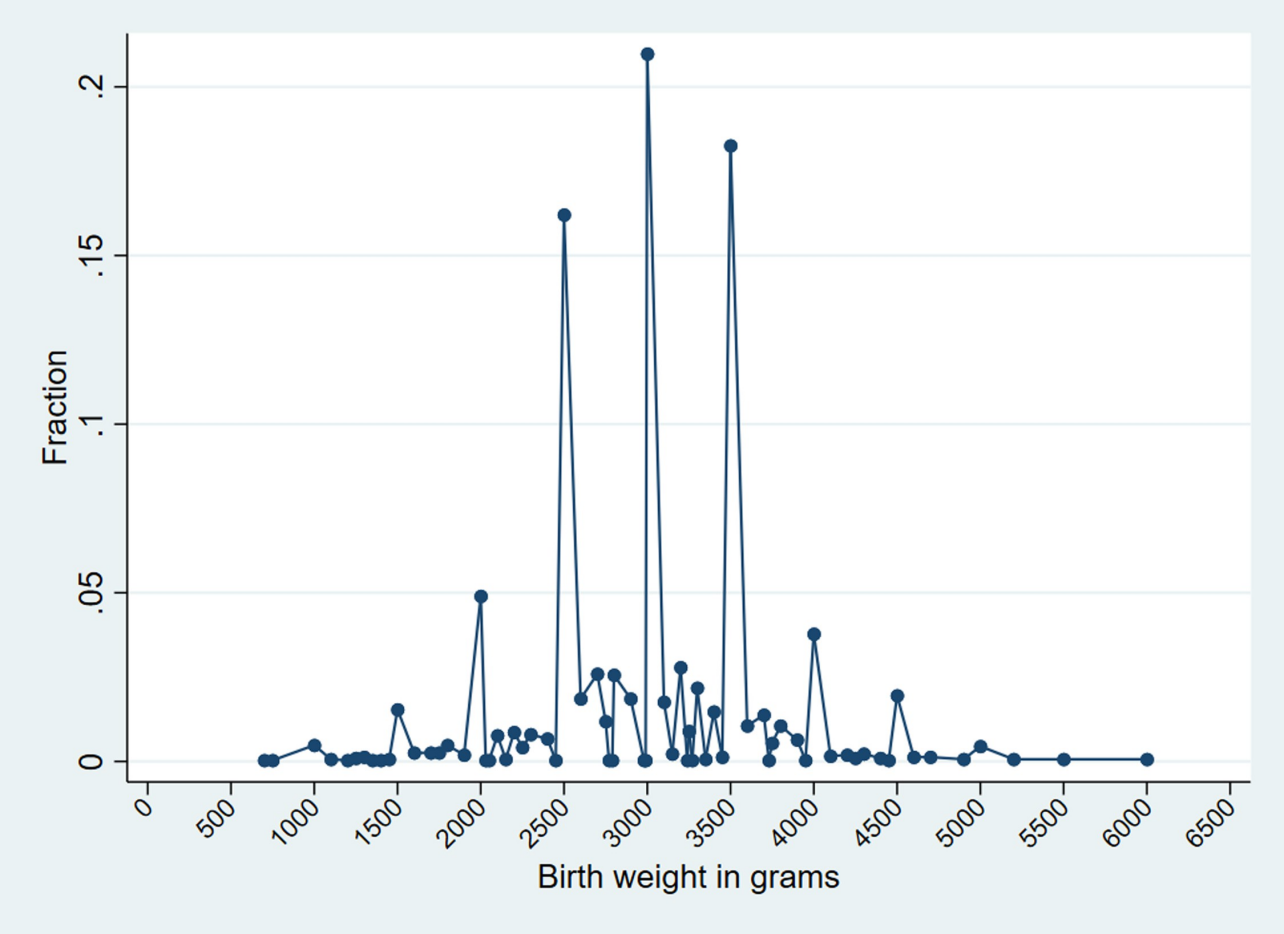
The fraction of newborn children according to their birth weight in grams, NDHS 2016.

Digit preference for the recording and recall of birth weight data was assessed. About 68.3% of respondents responded to birth weight as a multiple of 500 grams. Particularly, 16.1% of children were reported as having a birth weight of 2500 grams. Likely, about 21.5% and 17.7% reported having birth weights of 3000 grams and 3500 grams (Fig 1).

**Fig 2:**
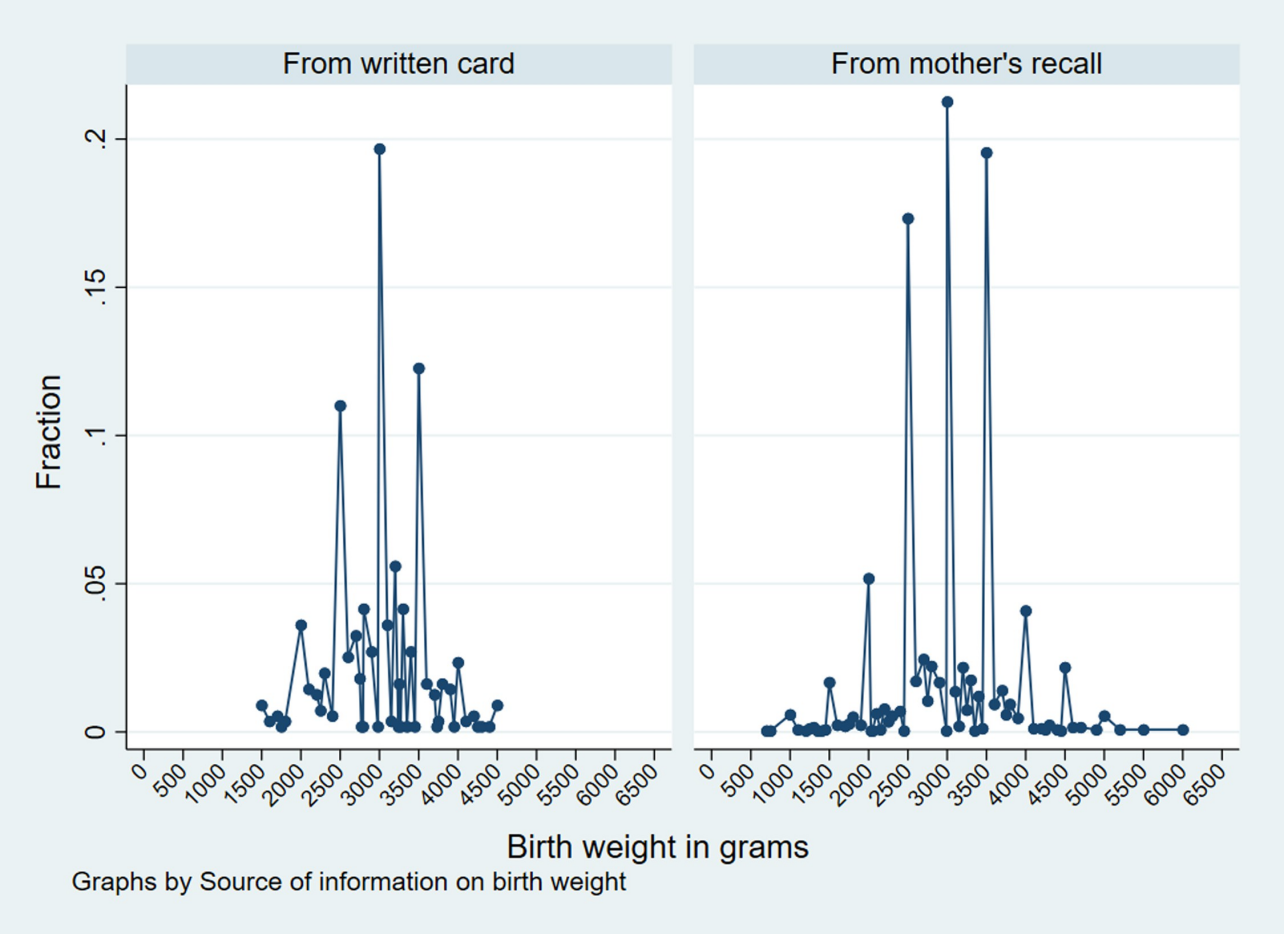
The fraction of newborn children according to their birth weight in grams obtained from written cards and mother’s recall, NDHS 2016.

Although a majority of data were heaped around the multiples of 500, there was no apparent difference in digit preference in the source of the birth weight data, i.e., collected from a written care or mother’s recall (Fig 2).

### Accuracy of the mother’s perception of baby size to predict LBW

The mean birth weight was 3002.9 grams (SD = 651.30). About 12.3% of babies weighed below <2500 grams, while about 16.3% of mothers perceived their babies as small at birth. Results further revealed that about 62% of the babies who had birth weight <2500 grams were perceived as small size at birth (smaller than average and very small) by their mothers (sensitivity = 61.99%). Similarly, among the babies with birth weight > = 2500 grams, about 90% were perceived as average or above-average size at birth (combined average, larger than average, and very large) by their mothers(specificity = 90.09%). Similarly, about 46.7% of the babies perceived smaller by their mothers at birth had birth weight <2500 grams (PPV = 46.68%). While 94.4% of the babies perceived average or above-average size at birth by their mothers had birth weight > = 2500 grams (NPV = 94.42%) (Table 2).

**Table 2:** Accuracy of mother’s perceived baby birth size to predict LBW, NDHS 2016 (N = 3095).

Variables	Birth Weight					
	<2500 g		> = 2500 g		Total	
	n	%	n	%	n	%
**Birth size$**						
Small#	236	7.61	269	8.70	505	16.31
Normal##	145	4.67	2446	79.02	2590	83.69
**Total (%)**	**380**	**12.28**	**2715**	**87.72**	**3095**	**100.00**
**Accuracy[95%CI]**						
Sensitivity	61.99	[55.44, 68.12]				
Specificity	90.09	[88.61, 91.39]				
Positive predictive value	46.68	[41.46, 51.98]				
Negative predictive value	94.42	[93.15, 95.47]				

$: derived from the variable Mother’s perception of size at birth.

#: combines two categories- smaller than average and very small.

##: combines three categories- very large, larger than average and average.

CI: confidence interval; PPV: positive predictive value; NPV = negative predictive value.

### Reliability of mother’s perceived baby birth size to predict LBW

The agreement between the mother’s perception of baby size (small and not small) and actual birth weight measurement (LBW and not LBW) was 86.3%, which was composed of a higher share of the agreement to identify non-LBW babies (79%) and a low share to identify LBW babies (7%). Also, there was a moderate agreement between the mother’s perception of size at birth and measured birth weight (Kappa = 0.45, 95%CI: 0.40, 0.51) (Table 3).

**Table 3:** Agreement among mothers’ perception of size at birth (small vs average or above average) and birth weight (<2500 grams vs. ≥ 2500 grams); NDHS 2016.

Statistic	Estimate	Standard Error	95%ConfidenceLimits		Observed Agreement
**Kappa statistic**	0.45	0.0177	0.40	0.51	86.32%

### Mother’s perception (5 categories) and normalized birth weight (5 categories)

There was only a fair agreement between five categories of the mother’s perceived birth size and normalized birth weight. The weighted kappa statistic was 0.39; (95%CI: 0.35, 0.43), and simple (unweighted) kappa was even lower (Kappa = 0.29; 95%CI:0.26, 0.33) (S3 Table).

The mother’s perception of the size of the baby at birth was concordant with the normalized birth weight among 67.1% of babies, while the mother’s perceived size at birth underestimated the normalized size at birth among 16.9% of babies and overestimated among 16.1% of babies (Table 1).

The percentage distribution of babies according to the mother’s perception of size at birth and normalized birth weight is shown in the S1 Fig.

The mean birth weight of babies perceived as very small at birth was about 2017 grams. As the perceived size moves higher, the mean birth weight was also significantly higher (p<0.001) (S4 Table). The babies perceived as very large also had the highest mean birth weight. The ANOVA model revealed that 33% (R-square = 0.33) of the variability observed in the birth weight is explained by the mother’s perceived birth size. A value of R-sq = 0.33 suggests that the mother’s perception of the birth size has a weak power to predict birth weight.

### Factors associated with the agreement between the mother’s perceived size of the baby at birth and the birth weight

Multivariable multinomial logistic regression analysis with concordant as the baseline category revealed that the mother’s education level, ethnicity, multiple births, and sex of the child were significantly associated with the underestimation or overestimation of weight at birth by the mother’s perception of the size at birth. On the other hand, place of residence, marital status, wealth quintile, age at delivery, four ANC visits, place of delivery, delivery by CS, birth order of the child, preceding birth interval, and unintended pregnancy variables were not significantly associated.

Compared to mothers without school education, mothers with primary school education were less likely to underestimate(OR = 0.69; 95%CI: 0.51, 0.95) than to report concordant size. Likely, mothers belonging to the relatively disadvantaged ethnicity were less likely to underestimate (OR = 0.64; 95%CI: 0.48, 0.85) and also less likely to overestimate (OR = 0.76; 95%CI: 0.59, 0.99) the birth weight than to report concordant size as compared to mothers from advantaged ethnicities (Table 4).

**Table 4:** Factors associated with discordance between mother’s perception of size at birth and birth weight, NDHS 2016, N = 3095.

	Mother’s estimation (base: concordant)			
Characteristics	Underestimation		Overestimation	
	OR	[95%CI]	OR	[95%CI]
**Mother’s education**				
No education	1.00		1.00	
Primary	0.69	[0.51, 0.95]*	1.10	[0.72, 1.67]
Secondary or above	0.91	[0.67, 1.24]	1.02	[0.72, 1.45]
**Ethnicity**				
Advantaged	1.00		1.00	
Relatively disadvantaged	0.64	[0.48, 0.85]*	0.76	[0.59, 0.99]*
Disadvantaged	1.40	[0.9, 2.17]	0.85	[0.57, 1.28]
**Child is twin**				
single birth	1.00		1.00	
1st of multiple	7.00	[2.14, 22.87]*	1.17	[0.28, 5.01]
2nd of multiple	3.66	[1.24, 10.81]*	0.55	[0.1, 2.93]
**Sex of child**				
Male	1.00		1.00	
Female	1.01	[0.8, 1.27]	0.77	[0.63, 0.95]*

Additionally adjusted for: the place of residence, marital status, wealth quintile, age at delivery, four ANC visits, place of delivery, delivery by CS, birth order of the child, preceding birth interval, and unintended pregnancy; not shown in the table as they were not statistically significant.

*p<0.05; OR = Odds Ratio; CI: Confidence Interval; NDHS: Nepal Demographic and Health Survey.

Similarly, mothers who had multiple (twin) births tend to underestimate their baby’s birth weight. Mothers were more likely to underestimate the size of multiple births for the first of multiple (OR = 7.00; 95%CI: 2.14, 22.87) and second of multiple births (OR = 3.66, 95%CI: 1.24, 10.81) than the reporting of concordant compared to single births. Similarly, mothers of female children were less likely to overestimate (OR = 0.77; 95%CI: 0.63, 0.95) the birth weight than the reporting of concordant compared to the mothers of male children (Table 4).

## Discussion

This study assessed the predictive accuracy of the mother’s perception of size at birth to the birth weight using the NDHS 2016 dataset. Among the low birth weight babies, only about 62% were correctly identified by the mother’s perception, while the agreement was moderate between the mother’s perception and birth weight regarding the detection of LBW. This study also found various socio-demographic and biological factors associated with the concordance of the mother’s perception of the baby’s size at birth to birth weight.

### Prediction of LBW

The sensitivity and positive predictive value of mothers’ perception to detect LBW were considerably low. This implies that the mother’s perception is not a strong indicator of detecting low birth weight in Nepal. It generates considerably high false-negative LBWs (about 38%). The specificity and negative predictive value of the mother’s perception to detect LBW were higher than the sensitivity and PPV. This implies that mothers correctly perceive large(no LBW) babies as large more frequently than the low birth weight babies as small babies. Higher specificity compared to sensitivity might contribute to the higher overall observed agreement (86.3%) between birth weight and the mother’s perceived size at birth. These findings suggest that mothers can predict more accurately for non-LBW babies (79% of the overall agreement 86.3%), but they are weak in identifying LBW (7.6%).

Another study utilizing the data from Ethiopian DHS found similar results regarding the predictive accuracy of the mother’s perception of size at birth and birth weight. That study reported a sensitivity of 57.0%, specificity of 88.6%, PPV = 41.3% and NPV = 93.6% [11]. Another study in Cameroon reported sensitivity = 60%, specificity = 93%, PPV = 44%, and NPV = 44% [19]. Another study from Oman reported a slightly lower sensitivity and specificity (sensitivity = 43.8%, specificity = 87.6%) but slightly higher PPV (54.9%) and similar NPV(94.4%) of mother’s perception of size at birth to predict the LBW, compared to the findings of this study [20]. These studies demonstrated that the mother’s perception has low sensitivity and PPV to detect LBW.

A hospital-based study in Nepal reported higher sensitivity of the mother’s perceived size at birth to predict LBW (sensitivity = 75%) compared to the findings of this study. However, the discrepancy in the value of sensitivity across these two studies could be due to the variation in the study methods [21]. Women undergoing hospital delivery are generally, more aware of health and risk associated with pregnancy, utilize more health services, including birth preparedness, ANC visit, and consultations, and are more knowledgeable about the birth outcome, including the size at birth than those of the general population of women of reproductive age and therefore the results are not comparable.

A multi-country study analyzing 62 nationally representative data from several countries between year 1990–2000 concluded that, on average, mothers’ perception generated 25% false-positive cases to detect LBW. The study recommended that instead of using the mother’s perceived size as the proxy of LBW, countries should promote the weighing of newborns [22].

### Overall agreement between the mother’s perception and birth weight

The overall agreement between the mother’s perception and birth weight five categories (unweighted kappa = 0.29 [95%CI:0.26, 0.33]), represents only a fair agreement. Nigatu D, *et al*. from Ethiopia reported similar findings [11]. These findings suggest that the mother’s perceived size at birth is a poor proxy for the LBW and birth weight.

### Determinants of discordance

This study identified that the concordance of the mother’s perception of size at birth and birth weight was associated with the mother’s education, ethnicity, multiple births, and the sex of a newborn child in Nepal.

Mothers with primary school education, compared to mothers with no school education, were less likely to underestimate birth weight compared to reporting concordance. This finding revealed that mothers with primary education are more accurate in relating the birth size based on birth weight compared to mothers without education. Another study from Nepal found that illiterate mothers were less likely to identify LBW than educated mothers [21]. Similar results were reported in Cameroon [19] and Uganda [23].

Mothers from relatively disadvantaged ethnicity were less likely to underestimate the birth weight of newborns compared to reporting concordance. This requires further study to explore why mothers from these ethnic groups tend to underestimate their baby’s birth weight.

Similarly, mothers who gave twin births were more likely to underestimate the birth weight than reporting concordant in comparison to mothers who gave birth to a single child. Multiple births are identified as a risk factor for LBW and childhood illness [24–26]. Therefore, mothers could have perceived their twin babies as vulnerable, weaker, and smaller than they are.

Interestingly, mothers of female children, compared to mothers of male children, were found significantly less likely to overestimate birth weight than reporting concordant. It could be partially attributed to the widespread perception that having a female infant is associated with LBW and having a male infant is associated with high birth weight. A qualitative study in Bangladesh, including mothers, and recently delivered women reported that the participants say that having a female child is a cause of LBW [25]. This perception might have made them accept that their newborn female baby is not large size.

The percentage of newborn babies weighed at birth has improved in Nepal from 36% in 2011 to 61% in 2016 [27]. However, it is still considerably low posing a major challenge to estimating the true burden of LBW in Nepal. Based on the available birth weight data, the prevalence of LBW was 12%, which did not change from 2011 [27]. Similarly, 9.3% of birth weight information was obtained from written cards in 2011 and 17.8% in 2016, which reveals that the birth weight data are heavily dependent on the mother’s recall which could be a source of recall bias or measurement error [13]. The low proportion of birth weight records could be associated with low institutional delivery rates in Nepal (57% in 2016) [13]. In our study sample, about 96% of institutional delivery mothers said that their baby was weighed at birth. In Nepal, like other developing countries, child feeding and caring practices are associated with a baby’s perceived birth size and birth weight. Normal birth weight babies are more likely to exclusively breastfeed and complete breastfeeding compared to babies with LBW [28]. Similarly, smaller size babies were less likely to have breastfed within 1 hour after birth [7, 29, 30]. When a baby’s birth weight is underestimated or overestimated by mothers or caretakers, babies’ health and nutritional status might be at risk due to these practices. Similarly, in Nepal, postnatal care visit within 2 days of delivery is still low (57% in 2016) [13]. In this scenario, there could be a long gap in the first contact between a recently delivered mother and a health worker, and in the absence of exact birth weight data, it is challenging for health workers to confirm the baby’s growth trajectory at an early stage, this could impact the child care plan and counseling that could be provided to mothers.

This study includes a large number of mother-children pairs from nationally representative surveys. DHS employs standard tools for data collection across the countries; therefore, the findings of this study are fairly generalizable and comparable locally, and globally.

This study has some limitations. The sample size included in the analysis is a fraction of the sample frame (3095/5060 = 61.2%) due to the missing value on birth weight. The characteristics of those who reported their birth weight significantly differed in various background characteristics. Therefore, this study could not rule out the possibility of selection bias. Also, data heaping and digit preference in birth weight data suggests the possibility of some measurement error in the birth weights. Notably, the spike in the frequency of women reporting birth weights 2500 grams, 3000 grams, and 3500 grams could be due to the rounding by the mothers or health workers when they didn’t know or recorded the actual birth weight might question the accuracy of the birth weight. This study could not eliminate the possibility of recall bias in the birth weight as only 17.8% of the birth weights were retrieved from actual record cards, while an overwhelming 82.2% of birth weight data were obtained from the mother’s recall.

Also, we compared the mother’s perception of the baby’s size with the birth weight. However, we aimed to estimate the predictive accuracy of baby size to birth weight, size could represent weight, length, or fat contents or a combination of these factors, which influenced the mother’s perception [6]. Information regarding these dimensions of size was not available in the survey data.

## Conclusion

This study found that the mother’s perception of birth size has low sensitivity and PPV to predict LBW in Nepal. Similarly, there is only a fair agreement between the mother’s perceived size and actual birth weight. The mother’s assessment of birth size somehow identifies babies with higher birth weights, but it lacks concordance in identifying LBW babies. These findings revealed that the mother’s perceived size of the baby at birth is a weak predictor of LBW. Furthermore, characteristics such as the mother’s education, ethnicity, multiple births, and sex of the newborn baby were significantly associated with the discordant birth size assessment. Therefore, when using a mother’s perception of size at birth to estimate LBW or birth weight, to reduce the chances of underestimation or overestimation, these background characteristics should be taken into consideration. To address the burden of LBW, efforts should be made to promote measuring and recording birth weight.

### Ethical approval

This study used publicly available secondary data from NDHS 2016. NDHS obtained ethical approval from Nepal Health Research Council Kathmandu, Nepal, and Macro Institutional Review Board, Maryland, USA [13]. NDHS obtained consent from mothers before starting the interview. We received permission from the Measure DHS program for further analysis [31]. Therefore, no separate ethical clearance was applied to this analysis.

## Supporting information

S1 Table Description of birth weight and mother’s perception of size at birth, 2016 (N = 3095). (DOCX)

S2 Table Characteristics of mothers and under five-year children with the percentage and odds of having weighed at birth in Nepal (NDHS 2016) N = 5060. (DOCX)

S3 Table Agreement among mother’s perception of size at birth (very small, smaller than average, average, larger than average, very large) and Normalized birth weight (five categories), NDHS 2016. (DOCX)

S4 Table Mean birth weight by mother’s perception of size at birth, NDHS 2016 (N = 3095). (DOCX)

S1 Fig Percent of newborn children according to mother’s perception and normalized birth weight, NDHS 2016. (PDF)
