# Supplementary material for: Mother’s perception of size at birth is a weak predictor of low birth weight: Evidence from Nepal Demographic and Health Survey
Source: PLoS One. 2023 Jan 24;18(1):e0280788. doi: 10.1371/journal.pone.0280788 (PMC9873179; doi:10.1371/journal.pone.0280788)
Supplement: S1 Table — (DOCX) [file pone.0280788.s001.docx]

**S1 Table. Description of birth weight and Mother's perception of size at birth, 2016, (N=3095)**

|  |  |  | **Agreement between weight and perception** | | | | | | |
| --- | --- | --- | --- | --- | --- | --- | --- | --- | --- |
| **Characteristics** | **Sample** | | **Concordant** | | **Underestimate** | | **Overestimate** | | **p-value** |
|  | n | **%** | **Row %** | **95% CI** | **Row %** | **95% CI** | **Row %** | **95% CI** |  |
| **Source of information** |  |  |  |  |  |  |  |  | p=0.056 |
| From written card | 551 | 17.81 | 71.62 | [66.95,75.87] | 15.87 | [12.40,20.09] | 12.51 | [9.79,15.86] |  |
| From mother's recall | 2544 | 82.19 | 65.99 | [63.77,68.14] | 17.03 | [15.29,18.93] | 16.98 | [15.39,18.70] |  |
| **Birth weight** |  |  |  |  |  |  |  |  | p<0.001 |
| <2500 gram | 382 | 12.35 | 43.05 | [37.45,48.83] | 42.22 | [36.81,47.83] | 14.73 | [11.02,19.41] |  |
| >=2500 gram | 2713 | 87.65 | 70.36 | [68.25,72.39] | 13.25 | [11.65,15.03] | 16.39 | [14.88,18.01] |  |
| **Mother's perception of size at birth** |  |  |  |  |  |  |  |  | p<0.001 |
| Very large | 116 | 3.74 | 21.43 | [13.70,31.92] | 78.57 | [68.08,86.30] | 0 |  |  |
| Larger than average | 433 | 13.98 | 25.66 | [20.94,31.03] | 64.42 | [58.67,69.79] | 9.92 | [7.22,13.48] |  |
| Average | 2039 | 65.87 | 87.45 | [85.78,88.94] | 6.1 | [5.08,7.32] | 6.45 | [5.36,7.75] |  |
| Smaller than average | 367 | 11.87 | 29.36 | [24.16,35.15] | 7.25 | [4.89,10.64] | 63.39 | [57.51,68.89] |  |
| Very small | 137 | 4.42 | 34.36 | [26.30,43.43] | 0 |  | 65.64 | [56.57,73.70] |  |
| don't know (n=4) | 4 | 0.12 | 0 |  | 0 |  | 100 |  |  |
| **Normalized birth weight** |  |  |  |  |  |  |  |  | p<0.001 |
| Very large | 103 | 3.34 | 23.96 | [15.54,35.06] | 0 |  | 76.04 | [64.94,84.46] |  |
| Larger than average | 238 | 7.69 | 46.65 | [39.52,53.92] | 11.87 | [8.05,17.17] | 41.48 | [34.66,48.63] |  |
| Average | 2392 | 77.3 | 74.52 | [72.30,76.61] | 13.95 | [12.20,15.92] | 11.53 | [10.17,13.04] |  |
| Smaller than average | 276 | 8.91 | 39.1 | [32.60,46.01] | 44.16 | [37.68,50.85] | 16.74 | [12.07,22.76] |  |
| Very small | 85 | 2.76 | 55.07 | [43.89,65.75] | 43.14 | [32.58,54.37] | 1.79 | [0.45,6.93] |  |
| **Birth size$** |  |  |  |  |  |  |  |  | p<0.001 |
| Small# | 505 | 16.31 | 30.72 | [26.28,35.54] | 5.29 | [3.56,7.79] | 64 | [59.09,68.63] |  |
| Average or above## | 2590 | 83.69 | 74.16 | [72.05,76.16] | 19.1 | [17.27,21.07] | 6.74 | [5.75,7.90] |  |
|  |  |  |  |  |  |  |  |  |  |
| **Total** | **3095** | **100** | **67.07** | **[65.08,69.01]** | **16.85** | **[15.26,18.56]** | **16.08** | **[14.67,17.60]** |  |

$: derived from the variable Mother's perception of size at birth

#: combines two categories- smaller than avearge and very small

##: combines three categories- verg large, larger than aveage and average
