# Supplementary material for: Mother’s perception of size at birth is a weak predictor of low birth weight: Evidence from Nepal Demographic and Health Survey
Source: PLoS One. 2023 Jan 24;18(1):e0280788. doi: 10.1371/journal.pone.0280788 (PMC9873179; doi:10.1371/journal.pone.0280788)
Supplement: S2 Table — (DOCX) [file pone.0280788.s002.docx]

**S2 Table. Characteristics of mothers and under five-year children with the percentage and odds of having weighed at birth in Nepal (NDHS 2016) N= 5060**

| **Characteristics** | **Sample** | | **Weighed at birth** | | **Having weighed at birth** | |
| --- | --- | --- | --- | --- | --- | --- |
|  | **N=5060** | **%** | **Row %** | **95% CI** | **OR** | **95%CI** |
| **Mother’s perception at birth** |  |  |  | **p=0.033** |  |  |
| very large | 175 | 3.45 | 66.27 | [56.83,74.58] | 1 |  |
| larger than average | 649 | 12.83 | 66.7 | [62.23,70.90] | 0.47 | [0.26, 0.84]* |
| average | 3363 | 66.47 | 60.68 | [58.78,62.55] | 0.41 | [0.24, 0.69]* |
| smaller than average | 629 | 12.43 | 58.49 | [54.01,62.83] | 0.28 | [0.16, 0.5]* |
| very small | 233 | 4.6 | 58.83 | [51.84,65.48] | 0.34 | [0.18, 0.66]* |
| don't know | 11 | 0.21 | 36.1 | [14.40,65.49] | 0.06 | [0, 0.83]* |
| **Place of residence** |  |  |  | p<0.001 |  |  |
| Urban | 2730 | 53.96 | 71.62 | [69.59,73.57] | 1 |  |
| Rural | 2330 | 46.04 | 49.07 | [46.83,51.32] | 0.84 | [0.68, 1.04] |
| **Mothers education** |  |  |  | <0.001 |  |  |
| No education | 1733 | 34.25 | 39.81 | [37.18,42.49] | 1 |  |
| Primary | 1019 | 20.14 | 54.84 | [51.34,58.29] | 1.43 | [1.08, 1.88]* |
| Secondary or above | 2308 | 45.61 | 80.16 | [78.28,81.91] | 1.65 | [1.26, 2.16]* |
| **Marital status** |  |  |  | P=0.118 |  |  |
| Single or unmarried | 39 | 0.77 | 73.09 | [57.70,84.39] | 1 |  |
| Married | 5021 | 99.23 | 61.15 | [59.58,62.69] | 0.29 | [0.1, 0.88]* |
| **Ethnicity** |  |  |  | p<0.001 |  |  |
| Brahmin/chhetri | 1396 | 27.59 | 74.67 | [72.33,76.88] | 1 |  |
| Janajati/Newar/Muslim/Others | 2969 | 58.68 | 57.24 | [55.10,59.36] | 0.45 | [0.35, 0.57]* |
| Dalit | 695 | 13.74 | 51.34 | [47.17,55.49] | 0.61 | [0.44, 0.85]* |
| **Wealth quintile** |  |  |  | p<0.001 |  |  |
| Poorest | 1082 | 21.39 | 41.63 | [38.74,44.57] | 1 |  |
| Poorer | 1072 | 21.19 | 50.62 | [47.16,54.08] | 1.02 | [0.77, 1.36] |
| Middle | 1121 | 22.16 | 60.87 | [57.54,64.09] | 2.04 | [1.5, 2.78]* |
| Richer | 1036 | 20.48 | 71.7 | [68.35,74.81] | 2.23 | [1.62, 3.06]* |
| Richest | 748 | 14.78 | 90.91 | [88.13,93.09] | 3.8 | [2.45, 5.89]* |
| **Four ANC visit** |  |  |  | p<0.001 |  |  |
| No | 2287 | 45.19 | 42.8 | [40.44,45.20] | 1 |  |
| 4 or more | 2773 | 54.81 | 76.44 | [74.64,78.15] | 1.91 | [1.54, 2.37]* |
| **Place of delivery** |  |  |  | p<0.001 |  |  |
| Non institutional | 2360 | 46.65 | 21.05 | [19.29,22.92] | 1 |  |
| Institutional | 2700 | 53.35 | 96.38 | [95.50,97.09] | 63.79 | [48.89, 83.22]* |
| **Mother's age at delivery** |  |  |  | p<0.001 |  |  |
| <20 years | 1280 | 25.29 | 66.36 | [63.41,69.19] | 1 |  |
| 20 or more | 3780 | 74.71 | 59.51 | [57.67,61.32] | 0.92 | [0.69, 1.23] |
| **Child is twin** |  |  |  | p0.902 |  |  |
| single birth | 4998 | 98.77 | 61.23 | [59.66,62.78] | 1 |  |
| 1st of multiple | 32 | 0.64 | 59.51 | [41.23,75.48] | 1.1 | [0.34, 3.58] |
| 2nd of multiple | 29 | 0.58 | 65.16 | [45.31,80.85] | 1.88 | [0.65, 5.46] |
| **Delivery by caesarean section** |  |  |  | p<0.001 |  |  |
| No CS | 4604 | 90.98 | 57.73 | [56.08,59.36] | 1 |  |
| CS delivery | 456 | 9.02 | 96.64 | [93.94,98.16] | 1.23 | [0.5, 2.99] |
| **Sex of Child** |  |  |  | P=0.132 |  |  |
| Male | 2647 | 52.32 | 62.38 | [60.24,64.47] | 1 |  |
| Female | 2413 | 47.68 | 59.99 | [57.70,62.24] | 0.85 | [0.69, 1.04] |
| **Birth order of child** |  |  |  | p<0.001 |  |  |
| First | 1993 | 39.39 | 77.77 | [75.65,79.75] | 1 |  |
| Second | 1479 | 29.22 | 61.66 | [58.73,64.51] | 0.66 | [0.49, 0.88]* |
| Third or more | 1588 | 31.39 | 40.12 | [37.48,42.82] | 0.57 | [0.41, 0.79]* |
| **Preceding birth space<24 months** |  |  |  | p<0.001 |  |  |
| No | 4408 | 87.11 | 63.89 | [62.24,65.51] | 1 |  |
| <24 months | 652 | 12.89 | 43.35 | [39.14,47.67] | 0.97 | [0.71, 1.32] |
| **Unintended pregnancy** |  |  |  | P=0.257 |  |  |
| Wanted | 4155 | 82.11 | 61.66 | [59.94,63.35] | 1 |  |
| Unwanted | 905 | 17.89 | 59.32 | [55.58,62.96] | 1.17 | [0.89, 1.54] |

*p<0.05; CI: Confidence Interval; NDHS: Nepal Demographic and Health Survey
