# Supplementary material for: Mother’s perception of size at birth is a weak predictor of low birth weight: Evidence from Nepal Demographic and Health Survey
Source: PLoS One. 2023 Jan 24;18(1):e0280788. doi: 10.1371/journal.pone.0280788 (PMC9873179; doi:10.1371/journal.pone.0280788)
Supplement: S3 Table — (DOCX) [file pone.0280788.s003.docx]

**S3 Table. Agreement among mother’s perception of size at birth (very small, smaller than average, average, larger than average, very large) and Normalized birth weight (five categories), NDHS 2016**

|  | **Actual measurements(normalized birth weights)** | | | | | |
| --- | --- | --- | --- | --- | --- | --- |
| **Perception of birth size by mothers** | **Very large** | **Larger than averate** | **Average** | **Smaller than average** | **Very small** | ***Total*** |
| **Very large** | 28 | 62 | 0 | 1 | 116 | ***116*** |
| **Larger than average** | 43 | 111 | 273 | 7 | 0 | ***433*** |
| **Average** | 36 | 96 | 1785 | 115 | 9 | ***2041*** |
| **Smaller than average** | 0 | 2 | 231 | 108 | 27 | ***368*** |
| **Very small** | 0 | 1 | 44 | 45 | 47 | ***137*** |
| ***Total*** | ***104*** | ***238*** | ***2394*** | ***275*** | ***84*** | ***3095*** |
|  |  |  |  |  |  |  |
| **Reliability statistics** |  |  |  |  |  |  |
| **Simple Kappa** | **0.29 (0.26, 0.33)** | | **67.18%** |  |  |  |
| **Weighted Kappa** | **0.39(0.35, 0.43)** | | **92.35%** |  |  |  |
