# Supplementary material for: Mother’s perception of size at birth is a weak predictor of low birth weight: Evidence from Nepal Demographic and Health Survey
Source: PLoS One. 2023 Jan 24;18(1):e0280788. doi: 10.1371/journal.pone.0280788 (PMC9873179; doi:10.1371/journal.pone.0280788)
Supplement: S4 Table — (DOCX) [file pone.0280788.s004.docx]

**S5 Table. Mean birth weight by mother's perception of size at birth, NDHS 2016, (N=3095)**

| **Mother's perception of size at birth** | **Mean birth weight in grams** | **[95% CI]** | |
| --- | --- | --- | --- |
| Very large | 3754.90 | 3594.54 | 3915.26 |
| Larger than average | 3534.85 | 3474.71 | 3594.99 |
| Average | 3012.93 | 2987.88 | 3037.98 |
| Smaller than average | 2451.27 | 2388.29 | 2514.25 |
| Very small | 2016.70 | 1903.49 | 2129.90 |
| **Mean birth weight** | **3002.91 (SD=651.30)** | **2976.91** | **3028.92** |

CI: Confidence interval; SD: Standard deviation
