# Supplementary material for: Mother’s perception of size at birth is a weak predictor of low birth weight: Evidence from Nepal Demographic and Health Survey
Source: PLoS One. 2023 Jan 24;18(1):e0280788. doi: 10.1371/journal.pone.0280788 (PMC9873179; doi:10.1371/journal.pone.0280788)

Percent of newborn children according to mother's perception and normalized birth weight, NDHS 2016

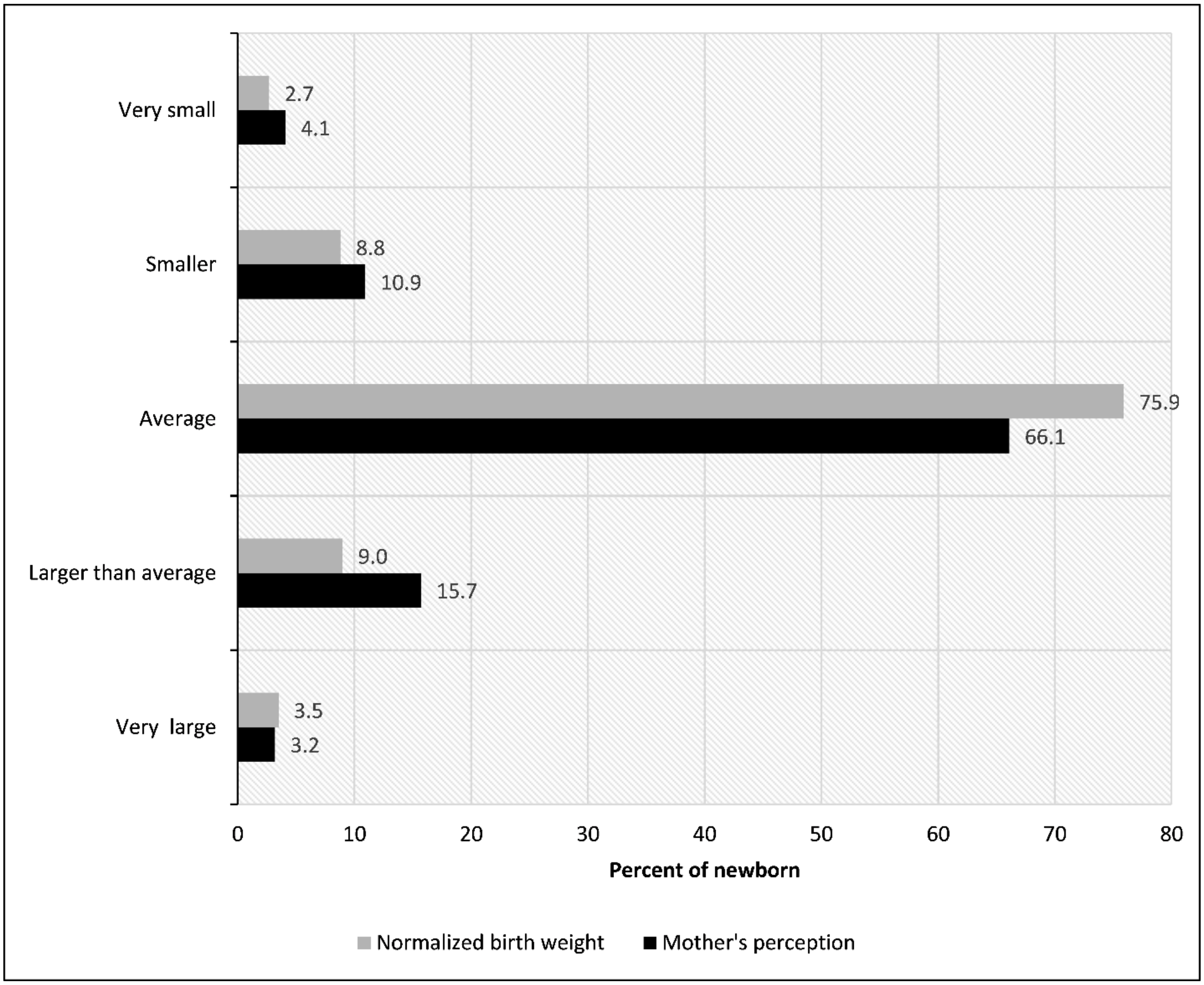

Supplement: S1 Fig — (PDF) [file pone.0280788.s005.pdf]
